# Preparation, Characterization and Application of Polysaccharide-Based Metallic Nanoparticles: A Review

**DOI:** 10.3390/polym9120689

**Published:** 2017-12-08

**Authors:** Cong Wang, Xudong Gao, Zhongqin Chen, Yue Chen, Haixia Chen

**Affiliations:** Tianjin Key Laboratory for Modern Drug Delivery & High-Efficiency, School of Pharmaceutical Science and Technology, Tianjin University, Tianjin 300072, China; 1015213012@tju.edu.cn (C.W.); xdgao@tju.edu.cn (X.G.); chenzhongqin@tju.edu.cn (Z.C.); chenyue0126@tju.edu.cn (Y.C.)

**Keywords:** biopolymers, polysaccharides, polysaccharide-based metallic nanoparticles, host–guest strategy, preparation and characterization, application, toxicity evaluation

## Abstract

Polysaccharides are natural biopolymers that have been recognized to be the most promising hosts for the synthesis of metallic nanoparticles (MNPs) because of their outstanding biocompatible and biodegradable properties. Polysaccharides are diverse in size and molecular chains, making them suitable for the reduction and stabilization of MNPs. Considerable research has been directed toward investigating polysaccharide-based metallic nanoparticles (PMNPs) through host–guest strategy. In this review, approaches of preparation, including top-down and bottom-up approaches, are presented and compared. Different characterization techniques such as scanning electron microscopy, transmission electron microscopy, dynamic light scattering, UV-visible spectroscopy, Fourier-transform infrared spectroscopy, X-ray diffraction and small-angle X-ray scattering are discussed in detail. Besides, the applications of PMNPs in the field of wound healing, targeted delivery, biosensing, catalysis and agents with antimicrobial, antiviral and anticancer capabilities are specifically highlighted. The controversial toxicological effects of PMNPs are also discussed. This review can provide significant insights into the utilization of polysaccharides as the hosts to synthesize MPNs and facilitate their further development in synthesis approaches, characterization techniques as well as potential applications.

## 1. Introduction

Scientific research on nanotechnology has received tremendous interest in this century due to its interdisciplinary applications in the field of catalysis, biomedicine, fuel cells, magnetic data storage and energy technology [1]. Nanoparticles (NPs), with the size range from 10.0 to 100.0 nm in diameter, have unique features including high surface area, quantum property as well as adsorption and releasing properties, exhibiting great potential in multifunctional applications [2]. Among all nanoparticles, metallic nanoparticles (MNPs) are especially attractive owing to their unique properties and diverse applications [3]. It has been accepted that the size, morphology, dispersibility and physicochemical properties of MNPs are strongly associated with their applications, which are affected by the synthesized approach [4]. Thus, the investigations of searching new hosts for controlling the properties of MNPs have been one of the main objectives in MNPs research [5]. Moreover, conventional synthesis techniques of MNPs are chemicals and energy consuming, causing various risks to the environment [6]. The awareness of developing an alternatively green synthesis approach is evolving into another objective in MNPs research [7]. To these aims, an ideal scheme is to emphasize these two objectives in parallel. 

**Figure 1:**
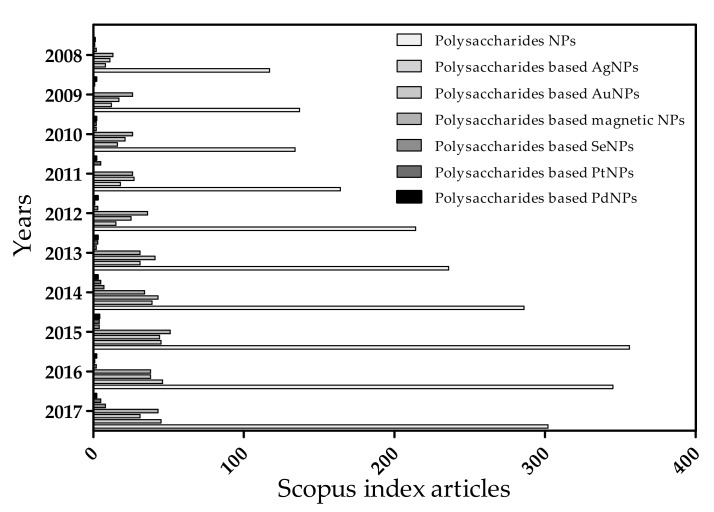
Scopus-indexed articles for polysaccharide based nanoparticles (NPs) and metallic nanoparticles (MNPs). (Archived until 6 November 2017).

Biopolymers are naturally abundant and environment friendly polymer alternatives, which are widely used in medical, agricultural and environmental industries due to their especially renewable, sustainable and nontoxic properties when compared to petroleum-based polymers [8]. According to the Food and Agriculture Organization (FAO, QC, Canada), around 35 million tons of natural fibers are harvested each year, which are a fundamental resource that can be used to produce biopolymers [9,10]. In the last decades, various biopolymers, including polysaccharides, proteins and nucleic acid, obtained from animals, plants and microbes, have been employed to packaging materials, drug delivery and regenerative medicine [11,12]. As significant types of biopolymers, natural polysaccharides in particular have some excellent properties owing to their chemical and structural diverse [13,14]. The differences ranging in charge, chain lengths, monosaccharides sequences, and stereochemistry give the highest capacity for the development of advanced functionalized materials and biomedicines [15]. Especially, the hydrophilic groups of polysaccharides can form non-covalent bonds with tissue cells and thus serve in cell–cell recognition and adhesion [16,17]. Moreover, for multi-gene, multi-step devastating disease such as cancer, diabetes and cardiovascular diseases, polysaccharides have a tendency to be more selectively to more than one specific site, eliminating the disadvantages of one-target strategy and preventing the over or under dosing of traditional delivery system [18,19]. Owing to the very safe and stable biodegradation, and biocompatibility, polysaccharides are considered as the most promising hosts in the synthesis of polysaccharide based metallic nanoparticles (PMNPs) with guest metallic ion and MNPs [20,21]. Besides, the host polysaccharides can assemble a carrier with metallic ion and hydrophobic chemical drugs such as doxorubicin, levofloxacin, cefotaxime, ceftriaxone, and ciprofloxacin for targeted drug delivery [22,23,24]. Polysaccharides can also act as good reducing and stabilizing agents to regulate the physical properties of PMNPs during synthesis process [25]. Over the last decades, remarkable progress has been achieved in the study of PMNPs through host–guest strategy (Figure 1) [26,27]. Therefore, it is necessary to review the recent progresses in PMNPs area. 

Although there are several relevant reviews about MNPs, reviews that introduce nanoparticles from the perspective of green synthesis of PMNPs through host–guest strategy are still limited [28]. In this review, a basic introduction of the utilization of natural polysaccharides as guest molecules, conjugated with the host molecules metal ions or MNPs to form PMNPs is given. First, we provide an in-depth introduction on the two common synthesis approaches and characterization methods. Then, the applications of PMNPs in different fields are summarized and discussed in detail. Finally, the potential toxic risks are demonstrated with both in vitro and in vivo evaluation, and future perspectives are presented. Patented PMNPs are not discussed in this review. 

## 2. Preparation of PMNPs 

It is widely accepted that the size, structure and corresponding physical, chemical and biological properties of nanoparticles are largely dependent on the preparation method [29]. Therefore, the selection of synthesis approach is crucial in achieving the appropriate properties of nanoparticles [30]. Generally, nanoparticles are prepared through a variety of chemical and physical methods, which bring serious problems such as high energy consumption, use of large amount of toxic solvents, and generation of hazardous byproducts [31]. Moreover, nanoparticles synthesized from chemical approach are not suitable in biomedical applications owing to the presence of toxic capping agents [32]. Alternatively, developing green synthesis approaches that use mild reaction conditions and non-toxic reaction precursors can eliminate the drawbacks of conventional approaches [33]. 

**Figure 2:**
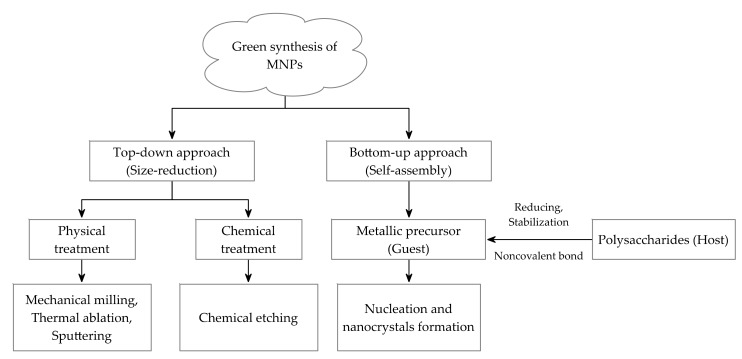
Overall approaches for the synthesis of PMNPs.

Green synthesis of MNPs, using natural polysaccharides as stabilizing and reducing agents, are considered to be a promising area in nanotechnology [34]. Currently, two approaches are involved in the green synthesis of nanoparticles: top-down and bottom-up approaches (Figure 2) [35]. In top-down synthesis, the suitable starting materials are reduced in size using physical treatments such as mechanical milling, thermal ablation or chemical treatment such as chemical etching [36]. After preliminary treatment, the surface of the MNPs will be altered and the high temperature and pressure during the size reduction may induce the oxidation of the nanoparticles, which can affect their physical properties and surface chemistry [35]. Therefore, bottom-up synthesis (self-assembly) is the most frequently chosen approach in the preparation of PMNPs. In a typical bottom-up synthesis, a metallic precursor is either decomposed or reduced to zero-valent state to form the building blocks (smaller entities), followed by the nucleation and nanocrystals growth [37,38,39]. During this process, polysaccharides can act as the hosts to combine with guest metallic ions and MNPs through noncovalent bonding, and then the order of free energy is altered to realize the stabilization, morphological control and kinetic growth of the MNPs [40]. In addition, the stereogenic centers of polysaccharides will also benefit for the anchoring of the MNPs [1]. In contrast to the harsh reaction condition of top-down approach, the bottom-up synthesis process can occur in the bulk solution or in droplets, which is easy to regulate through controlling the process conditions [41]. Therefore, PMNPs, synthesized through bottom-up synthesis approach that involved in host–guest strategy, are relatively homogeneous compared to those synthesized through top-down approach. 

Although PMNPs with different properties were achieved by regulating the temperature, reaction time and different molar ratios of polysaccharides and metal ions in most studies, there are some attempts that try improving the efficiency in the preparation of PMNPs [42]. The application of microwave heating in the synthesis of sulfated chitosan coated AuNPs resulted in a lower Gibb’s free energy and thus stimulated the activation of reaction [43,44]. Radiolytic reduction was also proven to be helpful in the nucleation, growth and aggregation of PMNPs during the synthesis process [45]. Owing to the different charges of metallic ions and polysaccharides in the solution, electrochemical synthetic techniques were considered to have great potential in the regulation of PMNPs synthesis. In addition, other techniques that could control the synthesis of PMNPs such as microemulsion and photoinduced reduction also need to be attempted in further study [46].

## 3. Characterization of PMNPs 

**Figure 3:**
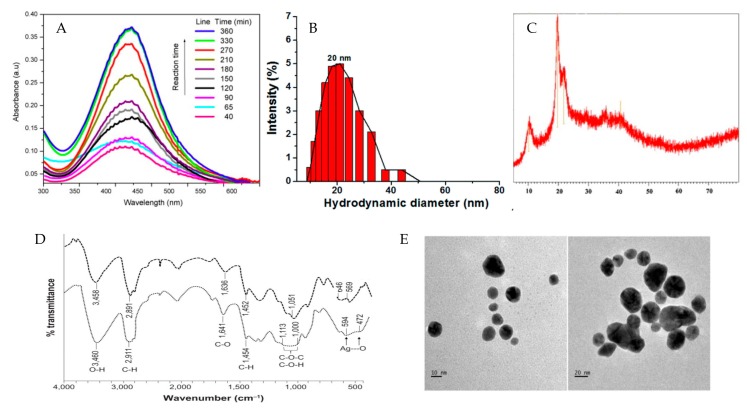
Overall scheme for the illustration of different methods to characterize PMNPs: (**A**) UV-vis spectra of AgNPs; (**B**) particle size distribution of AgNPs analyzed by DLS; (**C**) X-ray diffraction spectra of ZnSNPs; (**D**) FTIR spectra of AgNPs; and (**E**) TEM images of AuNPs. Reproduced with permission [23,43,65,66,67].

Nanoparticles are of great scientific interests as they combine bulk materials and atomic or molecular structures together [35]. These unique properties are largely associated with their physical characteristics such as diameter, molar volume, morphology and dispersibility [31]. Usually, nanoparticles have a spherical structure [47]. As for sophisticated nanoparticles, core–shell structure and particle distribution are also attractive for specific applications [48]. Thus, many techniques have been explored to elucidate the structure and spectroscopic characters of PMNPs in the literature (Figure 3). 

As the basic parameter of nanoparticles, size and morphology are firstly considered in many studies. Scanning electron microscopy (SEM) can give the information about the size, distribution and the shape of the nanoparticles [49]. However, the drying and contrasting process will alter the characteristics of nanoparticles, leading to imaging faults or artifacts [50]. Therefore, transmission electron microscopy (TEM) is introduced in the characterization of nanoparticles. It can be used to determine the particle size, dispersion and aggregation in aqueous environment with a high spatial resolution (<1.0 nm, Figure 3E) [51]. In addition, TEM can provide more details at the atomic scale such as crystal structure, which is more powerful and competitive than SEM [52]. However, many PMNPs are irregular in shape and thus hard to define. Moreover, they also tend to form large particles, and it is hard to attribute the reason to polydispersity or agglomeration [53]. In addition, it is difficult to measure the relevant size of some metallic colloids attached to drugs by TEM physiologically [49]. One technique that can solve this problem is dynamic light scattering (DLS), which can provide a measurement of particle size in solution [54]. In a DLS measurement, the nanoparticles will cause the fluctuations of the laser light intensity, which is recorded and used to determine the equivalent sphere hydrodynamic diameter of the particles [55]. DLS is also sensitive to flexible biological molecules, such as proteins and polysaccharides and thus suitable for the characterization of PMNPs [52]. Nevertheless, owing to the electron dispersion mechanism of DLS, it cannot provide information about the particle with heterogeneous size distributions [56]. Nanoparticles with similar sized distribution are also not well separated from each other in DLS measurement. Considering the diversity and ambiguity of nanoparticles, multiple techniques are commonly used to investigate nanoparticles size such as the combination of TEM and DLS [49]. 

UV-visible spectroscopy plays a significant role in the illustration of optical properties of PMNPs. It can monitor the quantitative formation and provide information for the size measurement of nanoparticles through different response to the electromagnetic waves, ranging from 190.0 nm to 700.0 nm [57]. The effects of concentration and pH on the stability and aggregation state of PMNPs in different time can also be recorded by UV-visible spectroscopy [35]. Fourier-transform infrared spectroscopy (FTIR) is basically applied to elucidate the functional groups from the spectrum. It can provide information about capping and stabilization of PMNPs, and therefore is utilized to demonstrate the conjugation between MNPs and polysaccharides [58]. X-ray diffraction (XRD) is the primary tool for the determination of the crystal property of PMNPs such as crystallite size and lattice strain [59]. Typically, XRD is useful for the characterization of the size and shape of crystalline PMNPs at the atomic scale. However, the requirement of single conformation and high atomic numbers of crystals limits the application of XRD [60]. In contrast to XRD, small-angle X-ray scattering (SAXS) can provide the information of the crystalline and amorphous PMNPs [51]. By analyzing the intensity of the X-ray, the size distribution, shape, orientation and structure of the nanoparticles can be well illustrated [61]. On the other hand, SAXS can give a holistic characteristic of the PMNPs, which is more effective than XRD. Nevertheless, the crystallite size of the PMNPs is not exactly the same as the particle size due to the polycrystalline aggregates of nanoparticles [62] and the lattice strain can provide detailed information about the distribution of lattice constant arising from crystal imperfections [63]. As for magnetic nanoparticles, a superconducting quantum interference device (SQUID) magnetometer is introduced to investigate their magnetization property, offering great insights into their application in specific field [64].

In addition to the above techniques, the number of novel methods for the characterization of PMNPs, such as scanning tunneling microscopy (STM), atomic-force microscopy (AFM), Raman scattering and electron spin resonance (ESR) spectroscopy, is growing rapidly, providing more evidence for their applications [35]. 

## 4. Application of PMNPs

Polysaccharide-based metallic nanoparticles have been extensively used in numerous technological fields owing to their remarkable physical, chemical and biological properties. Herein, the applications of PMNPs are introduced, specifically in wound healing, targeted delivery, biosensing, catalysis and agents with antimicrobial, antiviral and anticancer capabilities. 

### 4.1. Antimicrobial and Antiviral Property of PMNPs

Microbial infections are responsible for most common clinical diseases worldwide, bringing big threats to human public health [68]. Currently, the antimicrobial agents in the market are quaternary ammonium salt, metal salt solutions and antibiotics [33]. Unfortunately, the poor effectiveness and overuse of these agents have led to growing drug resistance of pathogenic bacterial and fungi strains, especially multidrug resistance strains [69]. Infections caused by these strains are more difficult to cure and prevent. Therefore, it is urgent to find novel antimicrobial agents with low toxicity and high efficiency or alternative therapies to solve these problems. Among all the candidates used for the treatment of bacterial infections, MNPs, especially AgNPs, have drawn much attention due to their small size, large surface to volume ratio and tunable plasmon resonance characteristics [70,71]. Since the 1920s, AgNPs were officially approved by the FDA administration to be used in wound therapy as an antibacterial agent; the exploration of MNPs in antibacterial field has increased rapidly [72]. 

**Figure 4:**
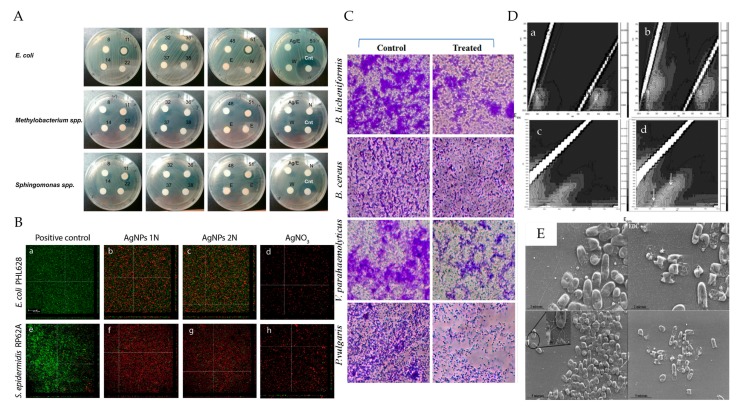
Overall scheme for the illustration of different methods to evaluate the antimicrobial activity of PMNPs: (**A**) antimicrobial tests on agar plate; (**B**) CLSM images of bacterial treated with p-AgNPs; (**C**) light microscopy images of bacterial treated with CS/Ag/ZnO nanocomposite; (**D**) fluorescent fingerprints of DNA of *E. coli* in the presence of exopolysaccharides based AgNP_S_; and (**E**) SEM images of bacterial treated with S-Chi@Au. Reproduced with permission [44,73,74,79,96].

Nowadays, numerous kinds of PMNPs have been synthesized and demonstrated to have significant antimicrobial potential (Table 1). Various methods had been applied to evaluate the antimicrobial activity of PMNPs (Figure 4). Among the methods, agar well diffusion method is the most widely used one to assess the antimicrobial activities of PMNPs against different bacteria and fungi due to its easy, quick and intuitive properties (Figure 4A). Results showed that AgNPs stabilized by different polysaccharides have effective antimicrobial effects on both Gram-positive bacteria, such as *S. epidermidis*, *S. aureus*, *S. lutea*, *B. subtilis*, *L. fermentum*, *E. faecium*, *B. licheniformis*, *B. cereus*, *K. rhizophila*, *S. pyogenes*, *Actinomycetes*, *Staphylococcus*, *S. pneumoniae*, *L. monocytogenes*, and *E. faecalis*, and Gram-negative bacteria, such as *E. coli*, *P. aeruginosa*, *K. planticola*, *K. pneumoniae*, *V. parahaemolyticus*, *P. vulgaris*, *S. typhimurium*, *A. hydrophila*, and *V. cholerae.* In addition, they also exhibited an extensively antifungal activity against *C. albicans*, *F. oxysporum*, *A. niger*, *T. rubrum*, *C. krusei*, and *A. flavus.* It appeared that different kinds of strains had different sensitivities toward polysaccharide-based AgNPs. Gram-negative bacteria were more likely to be affected due to their membrane compositions and the negatively charged cell wall, which made it easier to attach the released Ag^+^, which resulted in cell death [69]. Gram-positive bacteria were less susceptible to Ag^+^ compared to Gram-negative bacteria [33]. Confocal laser scanning microscopy (CLSM) was another method that used to assess the antimicrobial activities of polysaccharide-based AgNPs by measuring the fluorescence intensity of cells (Figure 4B) [73]. Besides, light microscopy could show the reduced biofilm on the glass surface and SEM could observe the changes in the shape and appearance of bacteria cells, providing more straightforward visualization choices (Figure 4C,E) [44,74]. It has been reported that the binding of Ag^+^ to oligonucleotides would cause changes in the fluorescence excitation and emission spectrum [72]. Thus, 3D fluorescence spectroscopy was introduced to investigate the interaction between Ag^+^ and DNA of bacteria (Figure 4D). Furthermore, minimum inhibitory concentration (MIC) is widely applied to evaluate the antimicrobial activity of PMNPs against bacteria and fungi that cultured in liquid medium [23].

Regardless of the fact that most reported PMNPs with antimicrobial activity were AgNPs, there are other PMNPs that exhibited great antimicrobial potential. AuNPs stabilized by bacterial exopolysaccharides and sulfate chitosan exhibited excellent antimicrobial properties, as assessed by the approaches of MIC, agar plate and SEM [25,75,76]. Polysaccharide-based SeNPs showed effective inhibitory activity on the growth of both bacteria and fungi in the agar plate [39]. In addition, starch copped CuNPs were also demonstrated with antimicrobial capability [77]. Nevertheless, the mechanisms underlying the antimicrobial effects of PMNPs remained unclear. Other proposed mechanisms such as interaction with enzymes and DNA, free radical production should be given more concern [33]. 

In the last decades, viruses have become serious concerns due to their dramatic breakout worldwide, including the human immunodeficiency virus (HIV), influenza virus, Zika virus and severe acute respiratory syndrome virus (SARS). The infection of virus has brought unprecedented crisis for public health, leading to a staggering societal cost [98]. Unlike the pathogenic microorganisms, viruses are infectious agents that replicate only in living cells, which can be eliminated by antibodies produced by the immune system instead of antibiotics [99]. The viral diversity and rapid mutability properties make the diagnosis and prevention of viruses especially difficult in the clinic [100]. PMNPs have posed various unique advantages, which make them attractive in solving the virus problems. It had been reported that tailored glycan functionalized AuNPs (13.0 nm) could bind with the influenza viruses (vieH5N1, shaH1N1 and H7N9) and form an aggregation on the surface of virus, resulting in a discriminable plasmon band shift and color change subsequently [100,101]. The polysaccharide-capped AgNPs (15.0 nm) were demonstrated to absorb and quench dye-labeled single-stranded DNA, which could be utilized as an effective fluorescence sensing platform for human immunodeficiency virus (HIV) [102]. In addition, polysaccharide-coated AgNPs (10.0 nm, 25.0 nm) were also illustrated to inhibit the viral replication and reduce the progeny production of Tacaribe virus and Monkeypox virus [103,104]. Nevertheless, the antiviral mechanism of polysaccharide-based MNPs is still unclear [105]. It is critical to understand the precise interactions between nanoparticles and virus, especially their influences on the binding of virus to host receptors, in further research. 

### 4.2. Anticancer Property of PMNPs

Cancer is a dominant factor of morbidity and mortality globally [106]. It has been estimated that about 90.5 million people are diagnosed with cancer, and the tendency is continually rising despite the preventive measures and therapeutic efforts in the past decades [107]. Recently, PMNPs, used in nanomedicine, provide an alternative opportunity in the prevention, diagnosis and treatment of cancer. Its sophisticated targeting strategies and multi-functional characters have remarkable advantages in the improvement of pharmacokinetics and pharmacodynamics profiles compared to conventional therapeutics [108]. Therefore, the application of PMNPs in cancer treatment as nanomedicine has emerged as a fruitful area in nanotechnology and extensive investigations have addressed this issue (Table 2). 

**Figure 5:**
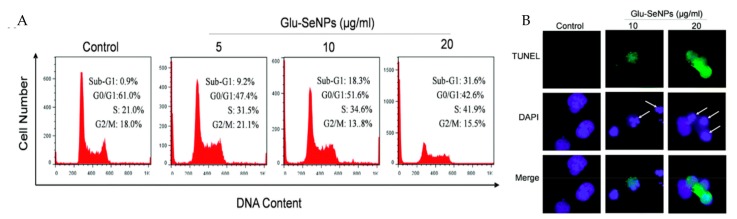
Apoptosis induced by Glu-SeNPs in cancer cells: (**A**) flow cytometric analysis of cancers cells; and (**B**) DNA fragmentation and nuclear condensation. Reproduced with permission [114].

3-(4,5-dimethylthiazol-2-yl)-2,5-diphenyltetrazolium bromide (MTT) assay is the most commonly used method to assess the effects of different toxicants on the viability of cells [109]. Pectin mediated AuPNs showed an obvious cytotoxicity on human breast adenocarcinoma cells MCF-7 and MDA-MB-231 with the concentration of 10 μg/mL via MTT reduction capability evaluation. Meanwhile, DNA of the cancer cells was also significantly fragmented indicating potential anticancer activity [110]. The noticeable alterations in the cellular shape and morphology were recognized as another hallmark of apoptosis of cancer cells [111]. It has been reported that chitosan based AgNPs (20.0 nm) reduced the viability of human lung adenocarcinoma cells A549/Lu, human hepatocellular carcinoma cells HepG2, human epidermic carcinoma cells KB and human breast carcinoma cells MCF-7 by reducing cell density and inducing the shrinkage and blebbing of the cells [23,112]. SeNPs have received additional attention in the prevention of cancers. Accumulated evidence illustrated that they could stimulate the immune system, modulate thioredoxin reductase activity, maintain cell redox balance and induce apoptosis of cancer cells [113]. D-glucose based SeNPs have been proven to exert anticancer activity against HepG2 cells at the cell cycle of S phase (Figure 5) [114]. In addition, absorption of polysaccharide-based SeNPs could also result in reactive oxygen species (ROS)-induced apoptosis of cancer cells through mitochondria mediated pathway [111]. 

Caspases-3 activation is another factor that involved in the execution phase of apoptosis [115]. Results showed that polysaccharide-based SeNPs and iron oxide NPs could significantly increase the activity of caspases-3 in a dose-dependent manner, resulting in the death of cancer cells [116,117]. Despite these results in the prevention of cancer cells, polysaccharide-based AuNPs were green synthesized to sufficiently eradicate tumors by means of heat and photothermal therapy [118,119]. In addition, the inhibition of *Tamarindus indica* polysaccharide-based AuNPs on tumor growth was also achieved by stimulating the proliferation of lymphocyte cells in vivo [111]. Therefore, PMNPs were good therapeutical agents to assist the cancer treatment. 

### 4.3. Wound Healing Property of PMNPs

Wound healing is a complex physiological process that follows three overlapping phases including inflammation and tissue remodeling, the rate of which is affected by the type, size and depth of the wound, and especially the presence of bacterial infection [128]. Consequently, conditions that are unfavorable for the colonization of pathogenic bacteria and fungus or helpful for the host repair mechanisms are required to facilitate wound healing progress [129]. Traditional dressings (gauze and tulle) achieve their capability of healing wound by forming a barrier from the external microorganisms and maintain the dry environment of the wound instead of influencing the healing process directly [130]. PMNPs had been extensively proved with excellent antimicrobial activity, providing great potential in the healing of wounds.

**Figure 6:**
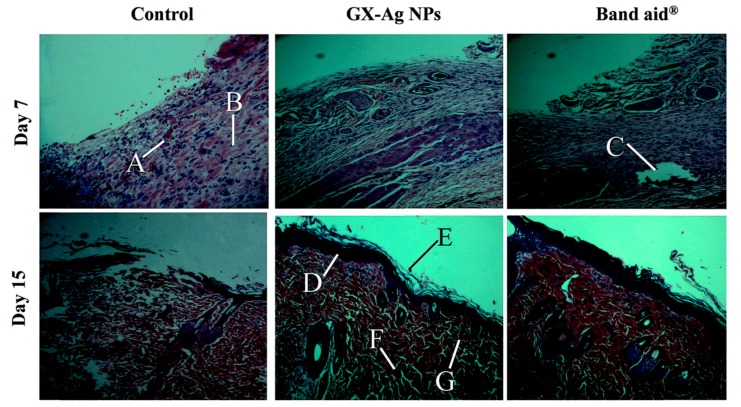
Histological examination in wound healing of glucuronoxylan-mediated AgNPs on Day 7, and 15 by Hematoxylin & eosin (H&E) staining: (**A**) neutrophils accumulation; (**B**) collagen deposition; (**C**) hair follicle; (**D**) epidermis; (**E**) stratum corneum; (**F**) dermis; and (**G**) fibroblasts. Reproduced with permission [137].

Nowadays, several PMNPs had been reported to be suitable for wound management. Pectin (8.0 nm) and chitosan (40.5 nm) coated AgNPs exerted great antibacterial activity on *E. coli* (Gram-negative) and *S. epidermidis* (Gram-positive) both on planctonic and biofilm formation conditions despite the low free Ag^+^ concentration [73,129]. It also exhibited cytocompatibility and capability in promoting fibroblasts proliferation through cytokine regulation [131]. Mannan sulfate AgNPs (20.0 nm) were reported to exhibit an enhanced cytocompatibility and promotion in the cellular uptake of murine macrophages, human skin fibroblasts and human keratinocytes. The excision and incision wound models in vivo also showed its repairs in wound area, wound contraction and epithelization period, suggesting promising potential for site-specific wound therapy [132]. In addition, xanthan based film incorporated with AgNPs could stimulate angiogenesis, which was the key factor of granulation tissue regeneration [133]. Nevertheless, more metallic nanoparticles preferred to use hydrogel as their reducing agents, which could be easily removed from the wound site, avoiding further trauma and facilitating re-epithelialization [134]. Usually, hydrogel mediated AgNPs revealed their wound healing ability through inhibition towards microorganisms [22,135,136]. Glucuronoxylan mediated AgNPs (9.3 nm) were proven to promote collagen content, which could stimulate the following re-epithelialization and granulation tissue formation (Figure 6) [137]. Recently, a novel bilayer composite was introduced in the wound dressing, which was more efficient than traditional single layer film. The upper layer, composed of chitosan based AgNPs, was designed to prevent bacterial penetration and ensured the permeability of oxygen to the wound site, while the lower layer, composed of cross-linked chitosan, was designed to improve cell proliferation activity [138]. This bilayer strategy had combined the advantages of both layers, which was an ideal strategy for the investigations of MNPs subsequently. 

### 4.4. PMNPs in Targeted Delivery 

Conventional chemotherapeutical agents have encountered various problems such as high toxicity, large volume of distribution, short lifetime in the body and low solubility, which led to a narrow therapeutical index [107]. Thus, it is critical to develop a directed therapy approaches and increase the efficiency of chemical agents. Nanoparticles represent unique physicochemical characters, which are small enough to traverse most biological membranes and avoid the uptake of the reticuloendothelial system [139,140]. These properties can be easily engineered with regard to the desired gene and drug delivery capacity and controlled release [141]. Over the years, PMNPs exhibited great potential in the targeted drug delivery applications and a summary of the current developments are shown in Table 3. 

One common targeted delivery application of PMNPs was to transport the anticancer drugs to the specific sites of cancer cells. It can be observed that the metallic nanoparticles that correspond to anticancer drug delivery are AuNPs, which exhibited a pH dependent release behavior, resulted in a higher release efficiency at pH 5.7 than pH 7.4 [142,143]. Generally, the endosomal pH of cancer cells is acidic, while the endosomal pH of normal cells is neutral [144]. This property will ultimately improve the cytotoxic activity of drugs against cancer cells and reduce their toxicity on normal cells, which is are desirable characteristics for cancer targeted drug delivery [145]. Besides, polysaccharide-based AuNPs revealed desirable optical, electrical and chemical properties, which enabled to display high-amplitude photoacoustic signals in the cancer cells, suggesting promising potential in photoacoustic image-guided drug release and synergistic chemo-photoacoustic therapy [142,146]. Much evidence suggests that there are many receptors on the cell surface, which are responsible for the interaction between PMNPs and the microenvironment around the cells [147]. Based on this knowledge, hyaluronic acid supported AuNPs and *Gracilaria lemaneiformis* polysaccharide-based SeNPs achieved their anticancer effects by recognizing the receptors of CD44 and α_v_β_3_ integrin in specific cancer cells, respectively [147,148], suggesting a new strategy for the design of targeted drug delivery. Another promising site-targeted type of MNPs was magnetic nanoparticles, which could be directed at the specific tissues by means of an external magnetic field and its significance in the anticancer drug delivery had been well discussed in the review [107]. 

Normally, biological therapeutical agents were susceptible to be hydrolyzed and digested in the gastrointestinal process and their low membrane permeability restricted the potential bioavailability in vivo [149]. Therefore, the possibilities of PMNPs as novel carriers for the delivery of biological agents were exploited. Recently, chitosan reduced AuNPs were demonstrated to enhance the transmucosal delivery of insulin and improve the pharmacodynamic activity [150]. PMNPs also exhibited a safe delivery of DNA in vivo, indicating great potential in gene therapy [151]. Nevertheless, the applications of PMNPs in the delivery of biological agents were still limited and more attempts should be given in this area. 

### 4.5. PMNPs for Biosensing 

A biosensor is an analytical device capable of converting a biological event into a physico-chemical signal, which is highly specific and efficient in a low detection limit for the analysis [161]. Nowadays, various biosensors for the detection of chemicals, metal ions, proteins and gas had been reported. PMNPs, had been intensively applied in biosensing field owing to their outstanding optical, electronic and chemical properties [162]. A summary of the current developments in biosensing and probes are shown in Table 4.

Among all the biosensors, optical sensors are the most promising candidates as they are sensitive, flexible and convenient [163]. The basic principles for optic sensors are based on the determination of colorimetric absorbance, reflectance, luminescence, refractive index and light scattering changes [164]. On this basis, some PMNPs had been developed as biosensors and probes. It had been demonstrated that alginate and dextrin based AgNPs showed a concentration dependent changes in the absorbance of manganese (II) and copper (II), separately, suggesting a good application in the detection of metal ions [165,166]. Conventional methods of detection of melamine are complex and time-consuming. Chitosan stabilized AuNPs could lead to a color change in the presence of melamine with the limited concentration of 6 × 10^−6^ g/L, providing an alternative way for onsite detection of melamine [167]. Ammonia is a toxic pollutant that threatens the health of human [168]. In this regard, ammonia sensors that could detect the ambient ammonia concentrations before damage occurs are necessary. Polysaccharide-based AuNPs and AgNPs had excellent performances in the sensing of ammonia at room temperature with the detection limit of 1 ppb [169,170] and guar gum based AuNPs could present a wider detection range of ammonia from 0.1 parts-per-quadrillion (ppq) to 75,000 parts-per-million (ppm) due to the variations in electrical conductivity [171]. Another toxic pollutant ia hydrogen peroxide, which could also be successfully monitored by optic H_2_O_2_ sensor (polysaccharide-based AgNPs) in the concentration range of 0.001 to 10 mM [163]. Tin oxide (SnO_2_), commonly known as semiconductor material, exhibited fast response, high sensitivity, low power consumption, mass-produced potency and wide operation working temperature range characteristics, which were especially suitable for the practical applications in gas sensors [172]. Therefore, polysaccharide-based Au-doped SnO_2_ nanoparticles have been bio-green synthesized and their high sensitivity in the sensing of NO_2_ and ethanol vapor make them possible agents for the monitoring of harmful gas [173,174]. In addition, MNPs coated with dextran provided possibilities in the measurement of amino acids and proteins such as cysteine and insulin with high sensitivity [175,176] and other applications in enzyme activity determination and large scale screening were also observed [177,178]. In some situations, fluorescent dyes could be introduced in the synthesis of PMNPs to improve their efficiency and this strategy could be an option for the future development of biosensors [179].

### 4.6. PMNPs in Catalytic Application 

**Figure 7:**
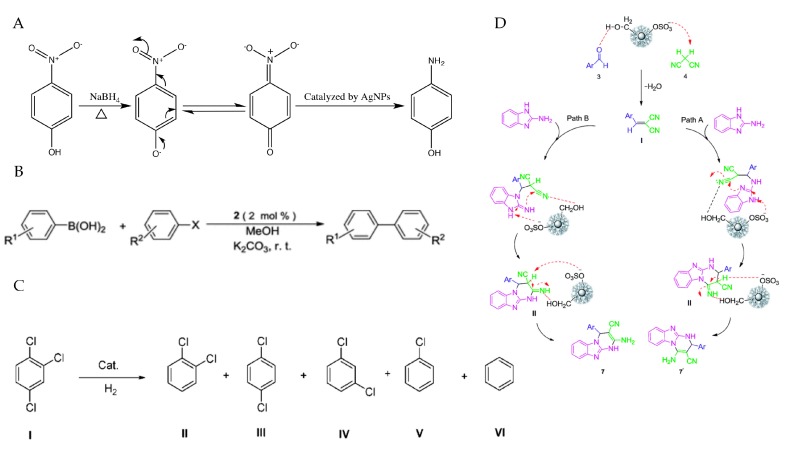
Overall scheme for the illustration of different catalytic reactions by PMNPs: (**A**) *4*-NP reduction catalyzed by arabinogalactan stabilized AgNPs; (**B**) Suzuki coupling reaction of aryl halide with arylboronic acid catalyzed by xylan-type hemicelluloses supported terpyridine-PdNPs; (**C**) hydrogenation of 1,2,4-trichlorobenzene catalyzed by *Klebsiella oxytoca* BAS-10 exopolysaccharides supported PdFeNPs; and (**D**) Synthesis of imidazopyrimidine derivatives catalyzed by Irish moss/Fe_3_O_4_ nanoparticles. Reproduced with permission [97,185,199,201].

Catalysis is an important process that associated with the chemical transformations, which is crucial for the development of modern industry [185]. As the key factors in catalysis, catalysts participate in the chemical reaction in a specific path without itself being consumed and they decide the rates of the reaction [1]. Ideally, catalysts can convert a large quantity of reactants, while consume less materials under mild reactive conditions [186]. However, traditional catalysts usually produce unwanted byproducts which have significant impacts on the environment [187]. In this regard, homogeneous catalysts that exhibited high selectivity had received much more attention. Among all the nanocatalysts, PMNPs are especially attractive due to their high surface area to volume ratios and high surface energy, which allowed their catalytic sites to be accessible [188]. Since catalytic reactions required transition metals, polysaccharides could provide a suitable functional support for dispersing the noble MNPs as hosts and make the size and shape of catalysts more controllable [1]. Thus, various studies had highlighted the possibilities of PMNPs in catalytic application (Table 5) and the reaction mechanisms were shown in Figure 7.

4-nitrophenol (4-NP) is a phenolic compound that widely exists in the wastewater [189]. It can cause serious damages to the central nervous system, kidney and liver in both animals and human beings [190]. Thus, various methods had been developed to reduce 4-NP to 4-aminophenol (4-AP), which was a common intermediate in the manufacture of antipyretic and analgesic drugs that friendly to the environment [191]. Commonly, the reduction of 4-NP to 4-AP was achieved in the presence of appropriate reducing agent named NaBH_4_ and the reaction could be monitored by UV-visible spectroscopy [192]. Thus, this model evaluation system was used to determine the catalytic activity of PMNPs. Nowadays, AgNPs and AuNPs stabilized by different kinds of polysaccharides were the most effective nano catalysts that reported to involve the conversion of 4-NP. *Cordyceps sinensis* exopolysaccharide-based AgNPs (5.0 nm) showed a good capability in the reduction of 4-NP with the activity factor of 15.75 s^−1^·g^−1^ [193]. AgNPs supported on xanthan gum (5.0–20.0 nm) could serve as a good catalyst for the reduction, and the large specific surface area of nanoparticles is favorable for the elevation of the efficiency [83]. *Portulaca arabinogalactan* stabilized AgNPs could facilitate electron transmission from BH_4_^−^ to 4-NP thereby stimulating the reaction [97]. AuNPs stabilized by Locust bean gum, glucomannan and katira gum also revealed excellent catalytic ability in the reduction of 4-NP to 4-AP with their first-order rate constants were 14.46 × 10^−2^ min^−1^, 6.03 × 10^−3^ s^−1^ and 2.67 × 10^−2^ min^−1^, respectively [174,194,195]. Moreover, biometallic Ag-Au nanoparticles capped by hydroxyethyl starch-*g*-poly (11.1 nm) were synthesized and exhibited great recyclability (98–93%) after 4 cycles, providing an enhanced efficiency in the 4-NP reduction reaction [196]. 

Suzuki–Miyaura reaction, involving the C–C bond formation, is widely used for the synthesis of many carbon molecules, which is one of the most significant cross-coupling reaction [197]. The PdNPs catalysts are really unmatched choices for this reaction [198]. Recently, xylan-type hemicelluloses supported terpyridine-PdNPs showed high catalytic activity and stability for Suzuki–Miyaura reaction between arylboronic acid and aryl halide under aerobic condition, with a yield of 98%. It also could be recovered conveniently and rescued at least six times without significant changes in their catalytic activity [199]. PdNPs (3.0 nm) could form an alginate matrix with Cu alginate gels and resulted in an improvement in the activity and recyclability of Suzuki–Miyaura reaction [200]. Besides, starch derived PdNPs (1.5–4.5 nm) were tested in the microwave-assisted Heck, Suzuki and Sonogashira C–C coupling reactions and excellent catalytic performances were observed, confirming the catalytic potential of the Pd-supported catalysts [133].

*Klebsiella oxytoca* BAS-10 exopolysaccharides supported PdNPs and PdFeNPs were investigated for catalyzing the hydrogenation of *trans-cinnamaldehyde* and 1,2,4-trichlorobenzene, respectively. Results showed that these two PMNPs could stimulate the hydrogenation under mild reaction conditions and their catalytic activities were maintained after several recycle experiments [201,202]. PMNPs also revealed remarkable catalytic ability in the degradation of dyes [203,204]. In addition, esterification of palm fatty acid distillate, benzylation of *o*-xylene, toluene and ethylene glycol oxidation as well as synthesis of imidazopyrimidine derivatives could also be catalyzed by different PMNPs [185,205,208]. Nevertheless, there still exist some problems in the isolation and recovery of these tiny nano catalysts from the reaction mixture through conventional methods due to their nano size and solvation properties [185]. The improvement of the recovery efficiency is obviously a significant objective in further research.

## 5. Toxicity of PMNPs

There is no doubt that polysaccharide-based metallic nanoparticles have made significant progress in many areas. Similar to most new technologies, the majority investigations in PMNPs have been focused on their potential applications and limited information is available on their toxicity to the cell, animals and the environment. Currently, some excellent review articles have provided significant insights into the toxicological significance and proposed mechanisms [217,218]. In fact, the risks associated with exposure to nanoparticles, the possible entry ways and metabolic mechanism have not been clarified until now, and several reports have illustrated that the nanoparticles deposit were responsible for toxic damages in different organs [219,220,221]. Although polysaccharides are attractive due to their low-toxic property and apparent lack of side effects [222], it is still necessary to evaluate the potential risks and toxicity of PMNPs in details. 

The major entry portals of PMNPs are respiratory system, oral ingestion, and skin absorption [204]. After being administered, the nanoparticles can be transported via blood circulation into different organs. It had been reported that the colloidal gold nanoparticles (30.0 nm) could be quickly observed in rat platelets after intratracheal instillation and Tc-labelled carbon particles (99.0 nm) could get into the blood circulation in 1 min [223,224]. Hemolysis, commonly known as the rupturing of red blood cells, can be induced by the oxidative-stress caused by nanoparticles, causing lysis and death of cells. Thus, the toxic effects of PMNPs were evaluated with reference to hemolysis percentage at first. Gum karaya-based AuPNs (15.0–20.0 nm) and xanthan-based AuPNs (20.0–25.0 nm) were found to display lower hemolysis rates, even at test concentrations over 200 μg/mL [155,225]. However, AgNPs (1.3–4.2 nm), use polysaccharides from edible mushroom *L. squarrosulus* (Mont.) Singer as the reducing and stabilizing agent, were found to be compatible with human red blood cells at a dose of less than 5 μg/mL [87]. Starch-based AgNPs (5.8 nm) could also induce significant hemolysis and exhibited a concentration-dependent in hemagglutination than AuNPs and PtNPs [226]. Therefore, the hemolysis risk assessment of PMNPs was needed before utilization. Other toxic effects of PMNPs were evaluated with the reference of cytotoxicity in normal cells. Accordingly, Gellan gum (10.0–15.0 nm) and Fucan (210.4 nm) coated AgNPs had a slight toxic effect towards to the mouse embryonic fibroblasts (NIH3T3) cells, human renal (HEK 293) cells and murine fibroblasts (3T3) cells [227,228]. Porphyran (14.0 nm) and pectin (7.0–13.0 nm) based AuNPs had no significant changes in normal monkey kidney cells viability, even up to 100 μM concentration [229,230]. In addition, chitosan coated CuNPs (260.0 nm) could obviously increase the viability of human alveolar epithelial (A549) cells relative to the exposure of CuNPs. Inflammatory risks induced by chitosan coated CuNPs would be raised if administered via the lung [231]. Normally, macrophages will participate in the clearance of nanoparticles that have passed the mucociliary barrier, which will lead to the activation of pro-inflammatiory mediators, causing both acute and chronic inflammation subsequently [232]. The investigations of inflammation in vitro, driven by PMNPs had showed that chitosan coated Ag/ZnO nanoparticles (100.0–150.0 nm) exerted no significant cytotoxic effects on murine macrophages RAW264.7 cells at the concentrations of 50 μg/mL and the cell morphology of cells was also not affected [74]. Additionally, PMNPs exhibited lower cytotoxicity than the metal itself, and different fluid exposure processes showed a significant effect on the viability of macrophage cells [233,234]. Several pieces of evidence also suggested that PMNPs had no neurotoxic effects and phyto-toxicity potential [235,236]. The sub-acute oral toxicity assessment also demonstrated the limited influences of PMNPs on the hematological and biochemical indexes of rats at the dose of 1500 ppm for 28 days [229,237]. Similar results were observed in the zebra fish toxicity study in vivo [238]. In addition, skin permeation study also showed that PMNPs could be detected in the receptor compartment in intact skin [227]. Although previous report had clarified that MNPs with the diameter smaller than 10.0 nm were able to penetrate the skin and reach the deepest layers of the stratum corneum, it did not permeate the skin [239]. 

Recent progresses in the toxicity investigation of PMNPs had put forward both the safety and risks at the same time. Nevertheless, it was still essential to consider the possible threaten that brought by MNPs in a long term exposure. Hence, more acute and sub-acute toxicity evaluation of PMNPs in vivo should be performed to avoid the potential hazards in the future. 

## 6. Conclusions and Perspectives

As the research in MNPs increases, the awareness towards seeking renewable multifunctional hosts for the preparation of MNPs has increased in both academia and society. Using natural polysaccharides exhibited enormous potential in the green synthesis of MNPs owing to their non-toxic, biocompatible and biodegradable advantages. Through host–guest strategy, polysaccharides can act as the reducing and stabilizing agents of metallic ions and MNPs, providing an alternative way of solving the problems in conventional physical and chemical synthesis approaches. The present review has focused on the recent advances in the preparation, characterization and application of PMNPs. For the synthesis of MNPs employing natural polysaccharides through bottom-up synthesis approach can not only consume few chemicals and energy, but also showed a good control of the size and morphology property of MNPs. Despite the extensive applications of various techniques in the characterization of PMNPs, the combination of multiple techniques is considered to be more suitable for illustrating their properties because of their diverse and ambiguous properties. The illustration of the characteristics of the PMNPs will provide more insights into the synthesis mechanism, which will be beneficial to the targeted synthesis of the nanoparticles. 

Although the development of green synthesis of PMNPs has achieved advanced progress, some problems still cannot be ignored. Due to significant variations in geography and time, the same species harvested in different seasons may lead to structural differences of the polysaccharides. Different synthesis methods of PMNPs also bring great challenges to their development and application. Thus, a commercially viable, eco-friendly and easy route for the synthesis of PMNPs is urgently needed. Another alternative attempt is to use synthetic polymers instead of the natural polysaccharides to synthesize PMNPs. The convenient management and adjustment of the synthetic process makes them to be a suitable host for the synthesis of homogeneous PMNPs. 

Primarily PMNPs and their application in specific fields including wound healing, targeted delivery, biosensing, catalysis and agents with antimicrobial, antiviral and anticancer capabilities are well demonstrated in detail. The applications of the PMNPs are largely associated with the characteristics of the metallic ions. Therefore, the guest metallic ions that exhibited good property in the field of optics, images, diagnosis and nanomedicine shall be considered in the synthesis of PMNPs. On the other hand, the controversial toxicological evaluations of PMNPs in recent years are introduced both in vitro and in vivo. Long-term toxicity investigations, such as for acute and sub-acute toxicity evaluation of PMNPs, are still required to elucidate their potential risks. Despite the remarkable advances, the recovery efficiency of PMNPs remains low. Further investigations that introduce physical methods such as the recovery of magnetic PMNPs through an external magnetic field to improve the recovery efficiency are urgently needed. In addition, different methods that involve host–guest strategy in the synthesis of PMNPs are also encouraged. 

## Figures and Tables

**Table 1:** Summary of literature data regarding the antimicrobial property of PMNPs.

Resource	Polysaccharides	Metals	Diameter (nm)	Shape	Antimicrobial Strains	References
*Lactobacillus plantarum*	Exopolysaccharides	Au	10.0–20.0	Spherical/ellipsoidal	*E. coli*, *S. aureus*, *K. pneumoniae*	[25]
*Pleurotus tuber-regium*	Polysaccharides-protein complexes	Se	122.0	-	*Staphylococcus*, *T. rubrum*	[39]
*-*	Hydroxypropylcellulose	Ag	25.0–55.0	Spherical	*E. coli*, *B. subtilis*, *S. aureus*, *P. aeruginosa*, *S. epidermidis*, *A. niger*, *Actinomycetes*	[43]
*-*	6-O-chitosan sulfate	Au	15.0	Spherical	*E. coli*	[44]
Tamarind	Carboxymethyl polysaccharides	Ag	20.0–40.0	Spherical/polygonal	*E. coli*, *B. subtilis*, *S. typhimurium*	[54]
*-*	Agarose/dextran/gelatin	Fe_2_O_3_	10.0	Dumbbell shape	*S. aureus*, *A. hydrophila*, *S. pyogenes*, *P. aeruginosa*	[64]
*-*	Guar gum	Ag	16.0	Spherical	*B. subtilis*	[65]
*-*	Chitosan-*g*-poly(acrylamide)	ZnS	19.0–26.0	Triangular	*E. coli*	[66]
*Astragalus membranaceus* root	Crude polysaccharides	Ag	65.1	Spherical	*S. aureus*, *E. coli*, *S. epidermidis*, *P. aeruginosa*	[69]
*-*	Pullulan	Ag	2.0–30.0	Spherical/ oval-shaped	*E. coli*, *K. pneumoniae*, *L. monocytogenes*, *P. aeruginosa*, *Aspergillus* spp., *Penicillum* spp.	[70]
*-*	Pectin	Ag	5.4–10.6	Spherical	*E. coli*, *S. epidermidis*	[73]
*-*	Chitosan	Ag/ZnO	10.0–65.0	Spherical/Uneven distribution	*E. coil*, *P. aeruginosa*, *L. fermentum*, *E. faecium*, *S. aureus*, *B. licheniformis*, *B. subtilis*, *B. cereus*, *V. parahaemolyticus*, *P. vulgaris*	[23,74]
*Bacillus megaterium*	Exopolysaccharides	Au	5.0–20.0	Spherical	*E. coli*, *B. cereus*, *S. aureus*, *S. epidermidis*, *K. pneumoniae*, *S. typhi*, *P. aeruginosa*, *V. cholerae*, *S. pneumoniae*	[75]
*Seaweed Chondracanthuschamissoi*, *LessoniaSpicata*, *Ulvasp*	Polysaccharides	Ag/Au	10.0/25.0	Spherical	*P. aeruginosa*, *S. typhimurium*	[76]
*-*	Starch	Cu(NO_3_)_2_	5.0–12.0	Spherical	*E. coli*, *S. aureus*, *Salmonella typhi*	[77]
*Padina tetrastromatica*	Fucoidan	Ag	17.0	Spherical	*B. subtilis, Bacillus* sp. *K. planticola*, *K. pneumoniae*, *S. nematodiphila, Streptococcus* sp.	[78]
*-*	β-glucan	Ag	15.0	-	*E. coli*, *Methylobacterium* spp., *Sphingomonas* spp.	[79]
*Arthrobacter sp. B4*	Exopolysaccharides	Ag	9.0–72.0	Face-centred-cubic	*P. aeruginosa*, *S. aureus*, *C. albicans*, *F. oxysporum*	[80]
*Cordyceps sinensis* (Berk.)	Exopolysaccharides	Ag	50.0	Spherical	*E. coil*, *S. aureus*	[81]
*-*	Xanthan gum/chitosan	Ag	5.0–20.0	Spherical	*E. coil*, *S. aureus*	[22,82,83]
*-*	Chitosan-carboxymethyl cellulose	Ag	5.0–20.0	Irregular shape	*E. coli*, *S. aureus*, *P. aeruginosa*	[84]
*Bradyrhizobium japonicum 36*	Exopolysaccharides	Ag	5.0–50.0	Rod/oval-shaped structures	*E. coli*, *S. aureus*	[85]
*Klebsiella oxytoca*	Exopolysaccharides	Ag	6.0–16.0	Spherical	*E. coli*, *K. rhizophila*	[86]
*Lentinus squarrosulus* (Mont.)	Hetero polysaccharides	Ag	1.3–4.5	Spherical	*E. coli*	[87]
*Pleurotus florida*	Glucan	Ag	1.3–2.5	Spherical	*K. pneumoniae*	[88]
Lactic acid bacterium	Exopolysaccharides	Ag	2.0–15.0	Spherical/triangular	*E. coli*, *K. pneumoniae*, *L. monocytogenes*, *P. aeruginosa*	[89]
*-*	Dextran/sucrose	Fe	5.8/7.3	Spherical	*E. coli*, *P. aeruginosa*, *E. faecalis*, *C. krusei*	[90]
*-*	Mesoporous starch	Ag	5.0–25.0	Spherical	*E. coli*, *S. aureus*	[91]
*Anogeissus latifolia*	Gum ghatti	Ag	5.5–5.9	Uneven shape	*E. coli*, *S. aureus*, *P. aeruginosa*	[92]
*Marine macro algae* (*U. faciata*, *P. capillacae*, *J. rubins*, *C. sinusa*)	Polysaccharides	Ag	7.0–20.0	Spherical	*E. coli*, *S. aureus*	[93]
*Bacillus subtilis*	Exopolysaccharides	Ag	1.1–6.7	Spherical	*S. aureus*, *P. aeruginosa*	[94]
*Cochlospermum gossypium*	Gum kondagogu	Ag	18.9–55.0	Spherical	*E. coli*, *S. aureus*, *P. aeruginosa*	[95]
*Porphyra vietnamensis*	Sulfated polysaccharides	Ag	10.0–16.0	Spherical	*E. coli*, *S. aureus*	[96]
*Portulaca*	Arabinogalactan	Ag	20.0–35.0	Spherical	*C. albicans*, *S. cerevisiae*, *A. niger*, *A. flavus*	[97]

**Table 2:** Summary of literature data regarding the anticancer property of PMNPs.

Resource	Polysaccharides	Metals	Diameter (nm)	Shape	Cancer types	References
*Tamarindus indica*	Galactoxyloglucan polysaccharides	Au	20.0	Spherical	Murine cancer cells (DLA, EAC)	[109]
*Musa paradisiaca*/ *Ganoderma lucidum*	Pectin	Au	8.0	Spherical	Human breast adenocarcinoma cells (MCF-7/MDA-MB-231)	[110]
*Tamarindus indica*	Polysaccharides PST001	Au	15.0–20.0	Circular	Breast cancer cells (MCF7), Leukemia cells (K562)	[111]
*-*	Fucoidan-mimetic glycopolymer	Au	20.0–55.0	Spherical	Human colon cancer cells (HCT116)	[112]
*Sargassum muticum*	Aqueous extract	Fe_3_O_4_	-	-	HepG2, MCF-7, HeLa, Jurkat	[116]
*Polyporus rhinocerus*	Polysaccharide–protein complexes	Se	50.0	Spherical	Human lung adenocarcinoma cells (A549)	[117]
*Halomonas maura*	Sulfated exopolysaccharides	Au	70.0–107.0	Quasi-spherical	Breast cancer cells (MCF7) Glioma cells (GI-1)	[118]
*-*	Gum arabic	Au	0.9–2.3	Spherical	Human breast adenocarcinoma cells (MDA-MB-231)	[119]
*Leuconostoc* spp.	Dextran	Au	49.0	Spherical	Ehrlich ascites carcinoma (in vivo)	[120]
*-*	Chitosan	Ag	5.0–15.0	Spherical	A549, HepG2, Lu, KB, MCF-7	[23,121]
*Chlorella vulgaris LARG-3*	Polysaccharides	Pt	18.0–38.0	Quasi-spherical	Ovarian cancer A2780	[122]
*Lentinus edodes*	Lentinan	Se	28.0	Spherical	Human cervix carcinoma cells (HeLa)	[123]
*-*	Hyaluronic acid	Se	66.8	Spherical	Heps solid tumor (in vivo)	[124]
*Undaria pinnatifida*	Polysaccharides	Se	59.0	Spherical	Human melanoma cells (A375)	[125]
*Spirulina*	Polysaccharides	Se	20.0–50.0	Spherical	Human melanoma cells (A375)	[126]
*Pleurotus tuber-regium*	Polysaccharide–protein complexes	Se	44.0–220.0	Spherical	Human breast carcinoma (MCF-7)	[127]

**Table 3:** Summary of literature data regarding the targeted delivery property of PMNPs.

Resource	Polysaccharides	Metals	Diameter (nm)	Shape	Targeted delivery	References
*Sphingomonas elodea*	Gellan gum	Au	12.0–14.0	Spherical	Doxorubicin hydrochloride delivery	[24]
*Lactobacillus plantarum*	Exopolysaccharides	Au	20.0–30.0	Spherical/ellipsoidal	Levofloxacin, cefotaxime, ceftriaxone, ciprofloxacin delivery	[25]
*-*	Mannan sulfate	Ag	17.0–23.0	Spherical	Targeting in cellular uptake (J774A.1, TE 353.Sk and HaCaT cells)	[132]
*Fucus vesiculosus*	Fucoidan	Au	73.0–96.0	Spherical	Doxorubicin delivery	[142]
*-*	Chitosan-oligosaccharide	Au	58.8–64.8	Spherical	Paclitaxel delivery	[143]
*-*	β-cyclodextrin-hyaluronic acid	Au	2.2	Spherical	Doxorubicin hydrochloride, paclitaxel, topotecan hydrochloride, camptothecin, irinotecan hydrochloride delivery	[144]
*-*	Poly(acrylamidoglycolic acid-*co*-vinylcaprolactam)-pectin	Ag	50.0–100.0	Spherical	5-fluorouracil delivery	[146]
*-*	Hyaluronic acid	Au	50.8–56.0	-	Binding with receptor CD44	[147]
*Gracilaria lemaneiformis*	Polysaccharides	Se	50.0	Near-spherical	α_v_β_3_ integrin receptor mediated endocytosis	[148]
*-*	Chitosan	Au	10.0–50.0	-	Insulin delivery, bioadhesive and intestinal barrier bypass characters	[150]
*Gynostemma pentaphyllum* Makino	Folate-conjugated sulfated polysaccharides	Au	4.0–6.0	Spherical	Camptothecin delivery	[152]
*Musa paradisiaca*	Gal-Glc-[Gal-]GlcNAc	Au	1.7–1.9	Spherical	Polysaccharides of Targeting in *Streptococcus pneumoniae* type 14	[153]
*-*	β-cyclodextrin/ chitosan	Fe	8.4–16.3	Spherical	Prodigiosin delivery	[154]
*-*	Gum karaya	Au	20.0–25.0	Spherical	Gemcitabine hydrochloride delivery	[155]
*-*	Dextran-lysozyme	Au	2.5–15.8	Spherical	Doxorubicin delivery	[156]
*Saccharomyces cerevisiae*	Mannan	Fe_3_O_4_	21.2–48.1	Ellipsoidal	Targeting in antigen-presenting cells/macrophage	[157,158]
*-*	Starch	Ag	11.5–19.3	Spherical	Targeting in mitochondrial membrane	[159]
	*k*-carrageenan	Fe_3_O_4_	4.0	Spherical	Methotrexate	[160]

**Table 4:** Summary of literature data regarding the polysaccharide-based MNPs for biosensing.

Resource	Polysaccharides	Metals	Diameter (nm)	Shape	Biosensing applications	References
*Ceratonia siliqua*	Locust bean gum	Au-SnO_2_/Ag	16.0–28.0	Spherical	Ethanol vapor sensing/hydrogen peroxide sensing	[163,164,174]
*-*	Alginate	Ag	10.0–20.0	Spherical	Detection of manganese (II) ions	[165]
*-*	Dextrin	Ag	15.0–28.0	Spherical	Detection of copper (II) ions	[166]
*-*	Chitosan	Ag/Au	7.3–8.8	Spherical	Detection of aromatic *ortho*-trihydroxy phenols/hydrogen sulfide/melamine	[167,180,181]
*-*	Guar gum	Au/Pd/Ag	6.0–10.0	Spherical	Sensor for the detection of ammonia level/electrocatalytic hydrazine	[169,171,182]
*Cyamopsis tetragonaloba*	Polysaccharides	Au	6.5	Spherical	Sensor for the detection of ammonia	[170]
*C. arietinum* L.	Water extracts	Au-SnO_2_	25.0	Spherical	Sensor for the detection of NO_2_	[173]
*Leuconostoc mesenteroides T3*	Dextran	Ag/Au	9.9–13.9	Spherical	Sensor for the detection of cysteine/insulin	[175,176]
-	Cellobiose	Au	10.7–33.5	-	Measurement of cellobiase activity	[177]
-	Hyaluronic acid	Au	14.0–19.0	Spherical	Hyaluronidase inhibitor screening	[178]
Bagasse	Xylan	Ag	20.0–35.0	Spherical	Detection of Hg^2+^	[183]
-	β-cyclodextrin-dextran-*g*-stearic acid	Fe_3_O_4_	59.0–149.0	Micelles	Magnetic resonance imaging for monitoring cancer cells	[184]

**Table 5:** Summary of literature data regarding the catalytic property of PMNPs.

Resource	Polysaccharides	Metals	Diameter (nm)	Shape	Reaction types	Reference
-	Xanthan	Ag	5.0–40.0	Spherical	*4*-NP reduction	[83]
*Portulaca*	Arabinogalactan	Ag	20.0–30.0	Spherical	*4*-NP reduction	[97]
*-*	Dextrin	Ag/Au	8.0–28.0	Spherical	*4*-NP reduction	[166]
*Ceratonia siliqua*	Locust bean gum	Au	-	Spherical	*4*-NP reduction	[174]
*Chondrus crispus*	Irish moss	Fe_3_O_4_	-	Homogeneous	Imidazopyrimidine derivatives synthesis	[185]
*-*	Alginate	Bi	5.0–8.0	Porous	*4*-NP reduction	[192]
*Cordyceps sinensis*	Exopolysaccharides	Ag	5.0	Spherical	*4*-NP reduction	[193]
*-*	Glucomannan	Au	12.0–31.0	Spherical	*4*-NP reduction	[194]
*Cochlospermum religiosum*	Katira gum	Au	6.9	Spherical	*4*-NP reduction	[195]
*-*	Starch-*g*-poly	Ag-Au	11.1	-	*4*-NP reduction	[196]
*-*	Xylan-type hemicellulose	Terpyridine-Pd	10.0–20.0	Particle	Suzuki–Miyaura reaction	[199]
*-*	Alginate	Pd-Cu	>10	Fibrils network	Suzuki–Miyaura reaction	[200]
*Klebsiella oxytoca* BAS-10	Exopolysaccharides	Fe/Fe-Pd	1.0–1.5	Cluster	Hydrodechlorination reaction	[201]
*Klebsiella oxytoca* BAS-10	Exopolysaccharides	Pd	30.0–550.0	Jagged undefined structures	Aqueous biphasic hydrogenation	[202]
-	Cellulose nanofibrils	Ag	25.2–18.0	Porous	Rhodamine B degradation	[203]
Corn	Crosslinked carboxymethyl starch/cellulose	ZnO/Zn	20.0–100.0	Spherical	Photodegradation of dyes	[204]
Algae	Algin	Al	4.0–5.0	Rough with wrinkled surface	Esterification reaction	[205]
*-*	Chitosan	ZrO	9.0	-	Benzylation of *o*-xylene	[206]
*-*	Sodium alginate	Cu-Mn	10.0–20.0	Spherical	Toluene oxidation	[207]
*-*	Dextrin	Au	8.4–12.0	-	Liquid phase oxidation of ethylene glycol	[208]
*-*	Chitin	Ag	5.5–15.2	Mesoporous, fibrous	*p*-NP reduction	[209]
*-*	Salep	Pd (II)	-	Rough	Suzuki coupling reaction	[210]
*-*	Alginate	Au	20.0–40.0	Centered cubic crystal lattice	Decoloration of Azo-Dyes	[211]
*Bupleurum falcatum*	Water extract	Au	8.2–12.8	Spherical	*4*-NP reduction	[212]
*Acetobacter xylinum* NCIM2526	Levan	Ag/Au	5.0–12.0	Spherical	*4*-NP reduction	[213]
*-*	Chitosan/ corn starch/ sodium alginate	ZnO	8.3–11.3	Hexagonal phase with Wurtzite structure	Photocatalytic reaction	[214]
*Pleurotus florida*	Glucan	Au	19.0–27.2	Spherical	*4*-NP reduction	[215]
*-*	Starch	Pd	1.5–4.5	Spherical	Heck reaction, Suzuki reaction, Sonogashira reaction	[216]

## Abbreviations

The following abbreviations are used in this manuscript: Metallic nanoparticles, MNPs; Polysaccharides based metallic nanoparticles, PMNPs; Confocal laser scanning microscopy, CLSM; Scan electron microscopy, SEM; Transmission electron microscopy, TEM; X-ray diffraction, XRD; small-angle X-ray scattering, SAXS; Scanning tunneling microscopy, STM; Dynamic light scattering, DLS; Atomic-force microscopy, AFM; Electron spin resonance, ESR; Minimum inhibitory concentration, MIC; Fourier transform infrared spectroscopy, FTIR; Reactive oxygen species, ROS; Human immunodeficiency virus, HIV; Severe acute respiratory syndrome, SARS; 3-(4,5-dimethylthiazol-2-yl)-2,5-diphenyltetrazolium bromide, MTT; Hematoxylin & eosin, H&E; *Escherichia coli*, *E. coli*; *Staphylococcus epidermidis*, *S. epidermidis*; *Staphylococcus aureus*, *S. aureus*; *Pseudomonas aeruginosa*, *P. aeruginosa*; *Sarcina lutea*, *S. lutea*; *Bacillus subtilis*, *B. subtilis*; *Klebsiella planticola*, *K. planticola*; *Klebsiella pneumoniae*, *K. pneumoniae*; *Candida albicans*, *C. albicans*; *Fusarium oxysporum*, *F. oxysporum*; *Lactobacillus fermentum*, *L. fermentum*; *Enterococcus faecium*, *E. faecium*; *Bacillus licheniformis*, *B. licheniformis*; *Bacillus cereus*, *B. cereus*; *Vibrio parahaemolyticus*, *V. parahaemolyticus*; *Proteus vulgaris*, *P. vulgaris*; *Salmonella typhimurium*, *S. typhimurium*; *Kocuria rhizophila*, *K. rhizophila*; *Aeromonas hydrophila*, *A. hydrophila*; *Streptococcus pyogenes*, *S. pyogenes*; *Aspergillus niger*, *A. niger*; *Trichophyton rubrum*, *T. rubrum*; *Vibrio cholerae*, *V. cholerae*; *Streptococcus pneumoniae*, *S. pneumoniae*; *Listeria monocytogenes*, *L. monocytogenes*; *Enterococcus faecalis*, *E. faecalis*; *Candida krusei*, *C. krusei*; *Saccharomyces cerevisiae*, *S. cerevisiae*; *Aspergillus flavus*, *A. flavus.*
